# Genomic Profiling Identifies Outcome-Relevant Mechanisms of Innate and Acquired Resistance to Third-Generation Epidermal Growth Factor Receptor Tyrosine Kinase Inhibitor Therapy in Lung Cancer

**DOI:** 10.1200/PO.18.00210

**Published:** 2019-03-27

**Authors:** Sebastian Michels, Carina Heydt, Bianca van Veggel, Barbara Deschler-Baier, Nuria Pardo, Kim Monkhorst, Vanessa Rüsseler, Jan Stratmann, Frank Griesinger, Susanne Steinhauser, Anna Kostenko, Joachim Diebold, Jana Fassunke, Rieke Fischer, Walburga Engel-Riedel, Oliver Gautschi, Eva Geissinger, Stefan Haneder, Michaela A. Ihle, Hans-Georg Kopp, Adrianus J. de Langen, Alex Martinez-Marti, Lucia Nogova, Thorsten Persigehl, Dennis Plenker, Michael Puesken, Ernst Rodermann, Andreas Rosenwald, Andreas H. Scheel, Matthias Scheffler, Werner Spengler, Ruth Seggewiss-Bernhardt, Johannes Brägelmann, Martin Sebastian, Bart Vrugt, Martin Hellmich, Martin L. Sos, Lukas C. Heukamp, Enriqueta Felip, Sabine Merkelbach-Bruse, Egbert F. Smit, Reinhard Büttner, Jürgen Wolf

**Affiliations:** ^1^ University Hospital of Cologne, Cologne, Germany; ^2^ Netherlands Cancer Institute, Amsterdam, the Netherlands; ^3^ University Hospital of Würzburg and Comprehensive Cancer Center Mainfranken, Würzburg, Germany; ^4^ Vall d'Hebron University Hospital, Barcelona, Spain; ^5^ University Hospital of Frankfurt, Frankfurt, Germany; ^6^ Pius Hospital Oldenburg and Lung Cancer Network NOWEL, Oldenburg, Germany; ^7^ University of Cologne, Cologne, Germany; ^8^ Cantonal Hospital Lucerne, Lucerne, Switzerland; ^9^ Lung Clinic Merheim and Hospitals of Cologne, Cologne, Germany; ^10^ University of Würzburg and Comprehensive Cancer Center Mainfranken, Würzburg, Germany; ^11^ Robert Bosch Centrum für Tumorerkrankungen, Stuttgart, Germany; ^12^ Private practice in Hematology and Oncology, Troisdorf, Germany; ^13^ University Hospital of Tübingen. Tübingen, Germany; ^14^ Sozialstiftung Bamberg, Bamberg, Germany; ^15^ University Hospital Zurich, Zurich, Switzerland; ^16^ Hematopathology Hamburg and Lung Cancer Network NOWEL, Hamburg, Germany

## Abstract

**PURPOSE:**

Third-generation epidermal growth factor receptor (*EGFR*) tyrosine kinase inhibitors (TKIs) are effective in acquired resistance (AR) to early-generation EGFR TKIs in EGFR-mutant lung cancer. However, efficacy is marked by interindividual heterogeneity. We present the molecular profiles of pretreatment and post-treatment samples from patients treated with third-generation EGFR TKIs and their impact on treatment outcomes.

**METHODS:**

Using the databases of two lung cancer networks and two lung cancer centers, we molecularly characterized 124 patients with *EGFR* p.T790M-positive AR to early-generation EGFR TKIs. In 56 patients, correlative analyses of third-generation EGFR TKI treatment outcomes and molecular characteristics were feasible. In addition, matched post-treatment biopsy samples were collected for 29 patients with progression to third-generation EGFR TKIs.

**RESULTS:**

Co-occurring genetic aberrations were found in 74.4% of *EGFR* p.T790-positive samples (n = 124). Mutations in *TP53* were the most frequent aberrations detected (44.5%; n = 53) and had no significant impact on third-generation EGFR TKI treatment. Mesenchymal-epithelial transition factor (*MET*) amplifications were found in 5% of samples (n = 6) and reduced efficacy of third-generation EGFR TKIs significantly (eg, median progression-free survival, 1.0 months; 95% CI, 0.37 to 1.72 *v* 8.2 months; 95% CI, 1.69 to 14.77 months; *P* ≤ .001). Genetic changes in the 29 samples with AR to third-generation EGFR TKIs were found in *EGFR* (eg, p.T790M loss, acquisition of p.C797S or p.G724S) or in other genes (eg, *MET* amplification, *KRAS* mutations).

**CONCLUSION:**

Additional genetic aberrations are frequent in EGFR-mutant lung cancer and may mediate innate and AR to third-generation EGFR TKIs. *MET* amplification was strongly associated with primary treatment failure and was a common mechanism of AR to third-generation EGFR TKIs. Thus, combining EGFR inhibitors with TKIs targeting common mechanisms of resistance may delay AR.

## INTRODUCTION

Treatment with selective early-generation epidermal growth factor receptor (EGFR) tyrosine kinase inhibitors (TKIs) has demonstrated high efficacy in patients with lung cancer harboring activating *EGFR* mutations. However, because of a Darwinian-like selection of drug desensitized tumor cells, resistance inevitably develops.^1-6^

In 60% of patients, acquired resistance (AR) is mediated through a mutation in the gate-keeper threonine of EGFR exon 20—p.T790M.^7,8^ Third-generation EGFR TKIs have been designed to overcome p.T790M-driven resistance, and confirmed response rates (RRs) range from 61% for osimertinib to 45% for rociletinib (CO-1686) and 55% for nazartinib (EGF816).^9-15^

Apart from monogenetically driven resistance, patients with tumor heterogeneity have been reported, including co-occurrence of p.T790M and amplifications of the mesenchymal-epithelial transition factor (MET) proto-oncogene (*MET*) or the human epidermal growth factor receptor 2 gene (*ERBB2)*, as well as mitogen-activated protein kinase/extracellular regulated kinase pathway activation.^17-25^ The combination of EGFR TKIs with other inhibitors may restore EGFR dependency and response to EGFR inhibition.^17-19,21-28^ Thus, the effects of co-occurring factors of resistance detected before third-generation EGFR TKI treatment and their impact on efficacy has been the focus of research.^19,24,25^ However, most reports are based on the analysis of cell-free DNA, and the numbers of matched pretreatment and post-treatment tumor samples are usually low. Apart from that, only a few studies have been performed that systematically investigated the impact of co-occurring aberrations on third-generation EGFR TKI outcomes. We present a comprehensive analysis of co-occurring genetic aberrations in pretreatment and post-treatment tumor tissue and their contribution to innate resistance (IR) and AR to three third-generation EGFR TKIs.

## METHODS

### Study Design, Patient Selection, and Tumor Tissue Collection

**FIG 1.**
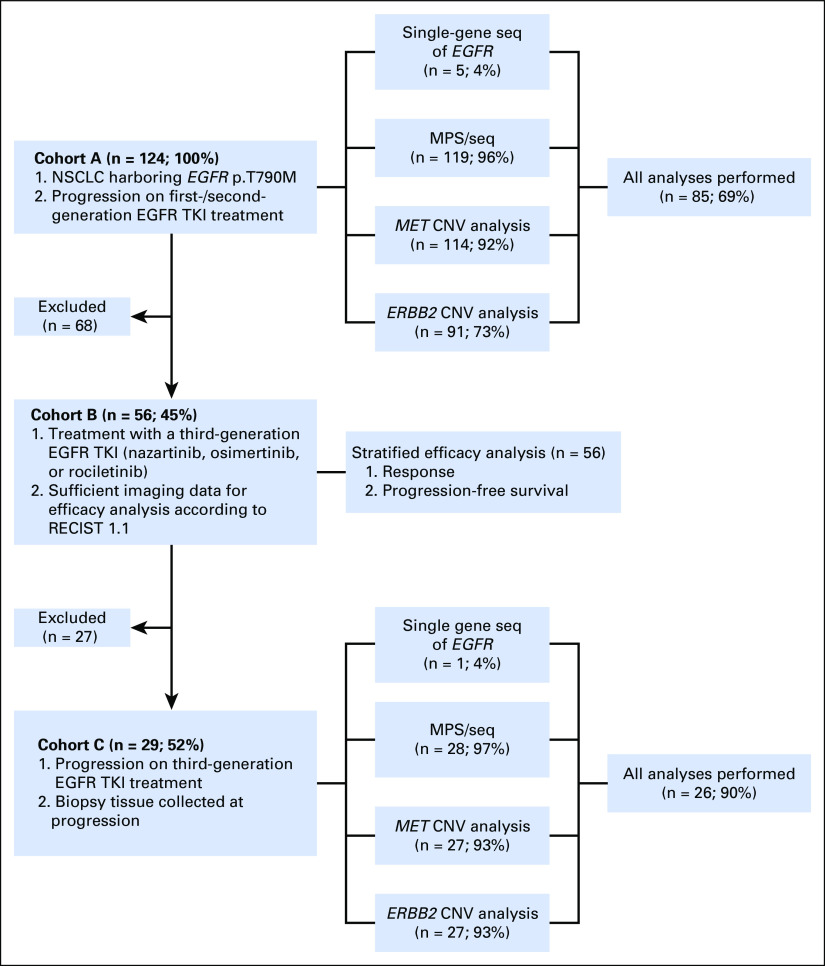
Flowchart of the study population and cohorts. CNV, copy number variation; *EGFR*, epidermal growth factor receptor; *ERBB2*, human epidermal growth factor receptor 2 gene; *MET*, mesenchymal-epithelial transition factor; MPS, massively parallel sequencing; NSCLC, non–small-cell lung cancer; RECIST, Response Evaluation Criteria in Solid Tumors; seq, sequencing; TKI, tyrosine kinase inhibitor.

To determine the frequency of co-occurring genetic aberrations in samples of *EGFR* p.T790M-mediated resistance to early-generation EGFR TKIs, we systematically searched the databases of the Network Genomic Medicine, the NOWEL network, the Department of Thoracic Oncology of the Netherlands Cancer Institute, and the Institute of Oncology at the Vall d’Hebron University Hospital for patients with non–small-cell lung cancer (NSCLC) who fulfilled the following selection criteria (cohort A; patients a1 to a68/b1 to b56; Fig 1; Data Supplement): (1) presence of *EGFR* p.T790M and (2) progression while receiving treatment with first- or second-generation EGFR TKIs.

To assess the effect of molecular aberrations on third-generation EGFR TKI efficacy in pretreatment and post-treatment samples, we selected patients from cohort A according to the following criteria (cohort B; patients b1 to b56; Fig 1; Data Supplement): (1) locally advanced/metastasized NSCLC harboring activating *EGFR* mutations and *EGFR* p.T790M, (2) third-generation EGFR TKI treatment in the setting of AR, and (3) sufficient imaging data for efficacy assessments according to Response Evaluation Criteria in Solid Tumors (RECIST) 1.1. Patients were treated in the AURA 1/3 trials (osimertinib; NCT01802632/NCT02151981), Tiger-2/-3 trials (rociletinib; NCT02147990/NCT02322281), CEGF816X2101 trial (nazartinib; NCT 02108964), osimertinib compassionate use program (CUP), or clinical routine. Patients treated in trials or the CUP were selected according to the specific eligibility criteria.

In a subset of patients from cohort B, a rebiopsy was performed at disease progression for identification of mechanisms of AR. These patients were grouped in cohort C (Fig 1).

In all patients, tumor tissue was collected in growing lesions by aspiration biopsy, core needle biopsy, or excisional biopsy (Data Supplement). All patients consented to the procedures according to local and Good Clinical Practice standards. Procedures were approved by the local ethics committees or review boards.

We identified three patients with *EGFR* p.G724S mutations (see Results). A more detailed description will be published elsewhere.^29^

### Efficacy Assessments

Patients treated within the osimertinib CUP or in clinical routine received scans as clinically indicated and per local practice. In patients treated within clinical trials, scans were performed according to the protocols. Scans were evaluated according to RECIST 1.1.^30^ Partial responses (PRs) were confirmed at least 4 weeks after the first scan showing a PR. IR was defined as progressive disease, (PD) as best response.

### Detection of EGFR p.T790M and Targeted Massively Parallel Sequencing

Tumor samples were formalin fixed paraffin embedded. Tumor tissue of patients was genomically characterized by massively parallel sequencing (MPS), if feasible. Four different MPS technologies and panels were used and are described in the Data Supplement in detail. In patients screened within Network Genomic Medicine (a1 to a68/b1 to b31/b37 to b43), MPS was performed with an Ion AmpliSeq Custom DNA Panel (Thermo Fisher Scientific, Waltham, MA) and a MiSeq benchtop sequencer (Illumina, San Diego, CA) or with a GeneRead DNAseq Custom Panel V2 (Qiagen, Santa Clarita, CA) consisting of 205 amplicons.^31^

In patients screened within the NOWEL network (a33 to a36), sequencing was performed using the NEOPlus hybrid-capture–based approach (NEO New Oncology, Cologne, Germany). Samples of patients from the Netherlands Cancer Institute (b44 to b56) were analyzed on a MiSeq benchtop sequencer (Illumina) using the TruSeq Amplicon Cancer Panel v1.0 (Illumina). For patients in which MPS was not feasible, *EGFR* status was determined by Sanger sequencing or digital droplet polymerase chain reaction. The molecular analyses performed in each sample are available in the Data Supplement.

### Determination of Copy Number Variations and Small-Cell Lung Cancer Transformation

*MET* copy number variation (CNV) analysis was performed by fluorescence in situ hybridization using the ZytoLight SPEC *MET/CEN7* Dual Color Probe (ZytoVision, Bremerhaven, Germany).^20^ Samples were classified as *MET*-amplified if fulfilling the criteria for high-level amplification established by Schildhaus et al^20^ (ie, *MET/CEN7* ratio greater than or equal to 2.0 or an average *MET* gene copy number [GCN] per cell of greater than or equal to 6.0).^23^ All other tumors were classified as *MET* wild type (WT).

*ERBB2* CNV status was determined using the ZytoLight SPEC *ERBB2*/*CEP17* Dual Color Probe (ZytoVision) or the INFORM HER2 Dual ISH DNA Probe (Ventana, Tucson, AZ).^17^ Amplification of *ERBB2* was positive if the *ERBB2/CEP17* ratio was greater than or equal to 2.0 or the average *ERBB2* GCN per cell was greater than or equal to 6.0. In the post-treatment samples (cohort C) of b41 to b56, *MET* and *ERBB2* status was assessed by fluorescence in situ hybridization or chromogen in situ hybridization only if CNVs were detected by MPS.

Small-cell lung cancer transformation was assessed using microscopy by experienced pathologists. Transformation was defined by the occurrence of small-cell lung cancer histology.

### Statistical Analyses

RR was defined as the percentage of complete remissions and PR as best response. Progression-free survival (PFS) indicated the time from treatment start until PD or death. Overall survival (OS) was defined as the time from first diagnosis until death. Time-to-event end points were analyzed using the Kaplan-Meier estimator. Qualitative variables were summarized by count and percentage; quantitative variables were summarized by mean, median, and range. Differences in time-to-event distribution were evaluated by the log-rank test, and statistical association between any two categorical variables was assessed by Fisher’s exact test; 95% CIs for proportions were calculated using the Clopper-Pearson (binominal) formula. *P* values less than or equal to .05 were considered statistically significant. The frequencies of the genetic changes were calculated on the basis of the number of patients screened for each aberration. Calculations were performed in Excel (Microsoft, Redmond, WA) and SPSS Statistics version 24 (SPSS, Chicago, IL).

## RESULTS

### Clinical and Molecular Characteristics of Patients With p.T790M-Positive AR to Early-Generation EGFR TKI Therapy (cohort A) and Impact on Outcome of Third-Generation EGFR TKI Treatment (cohort B)

The molecular characteristics of cohort A (n = 124) and the impact on OS are illustrated in the Data Supplement. A total of 56 patients (45%) from cohort A fulfilled the selection criteria for cohort B and showed the clinical characteristics outlined in the Data Supplement. Patients received third-generation EGFR TKI treatment with osimertinib (n = 37; 66.1%), nazartinib (n = 11; 19.6%), and rociletinib (n = 8; 14.3%).

The RR in the overall population was 61% (95% CI, 46.8% to 73.5%), and median PFS was 8.0 months (95% CI, 6.9 to 9.1 months; Table 1). Efficacy of osimertinib and nazartinib treatment was not significantly different. One PR was confirmed while the patient was taking rociletinib, and RR was 12.5% (95% CI, 0.3% to 52.7%). Median PFS with rociletinib was 3.7 months (95% CI, 0.0 to 7.9 months).

**TABLE 1.**
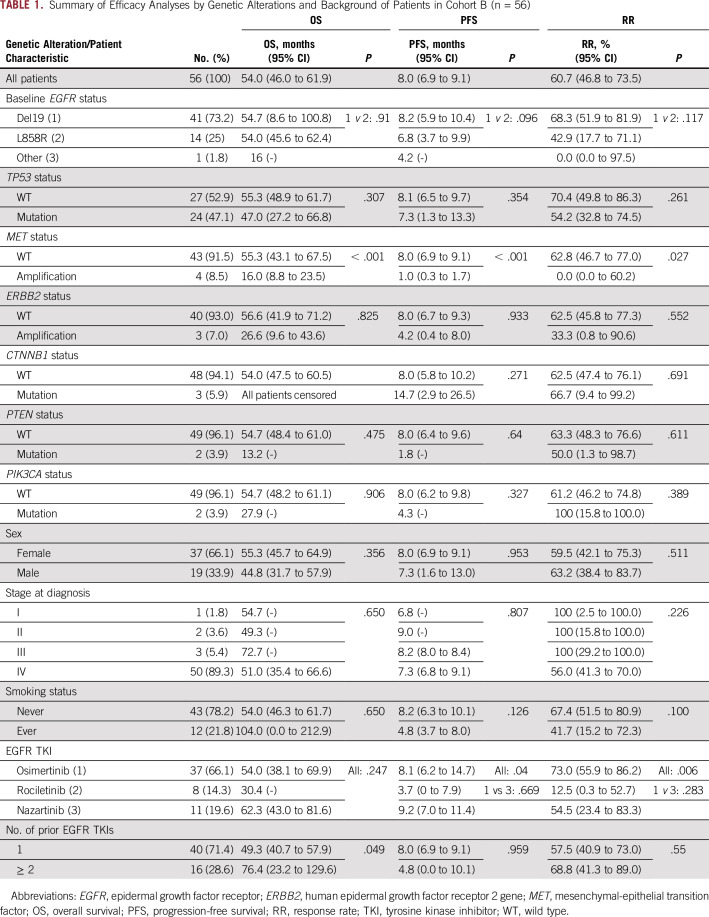
Summary of Efficacy Analyses by Genetic Alterations and Background of Patients in Cohort B (n = 56)

**FIG 2.**
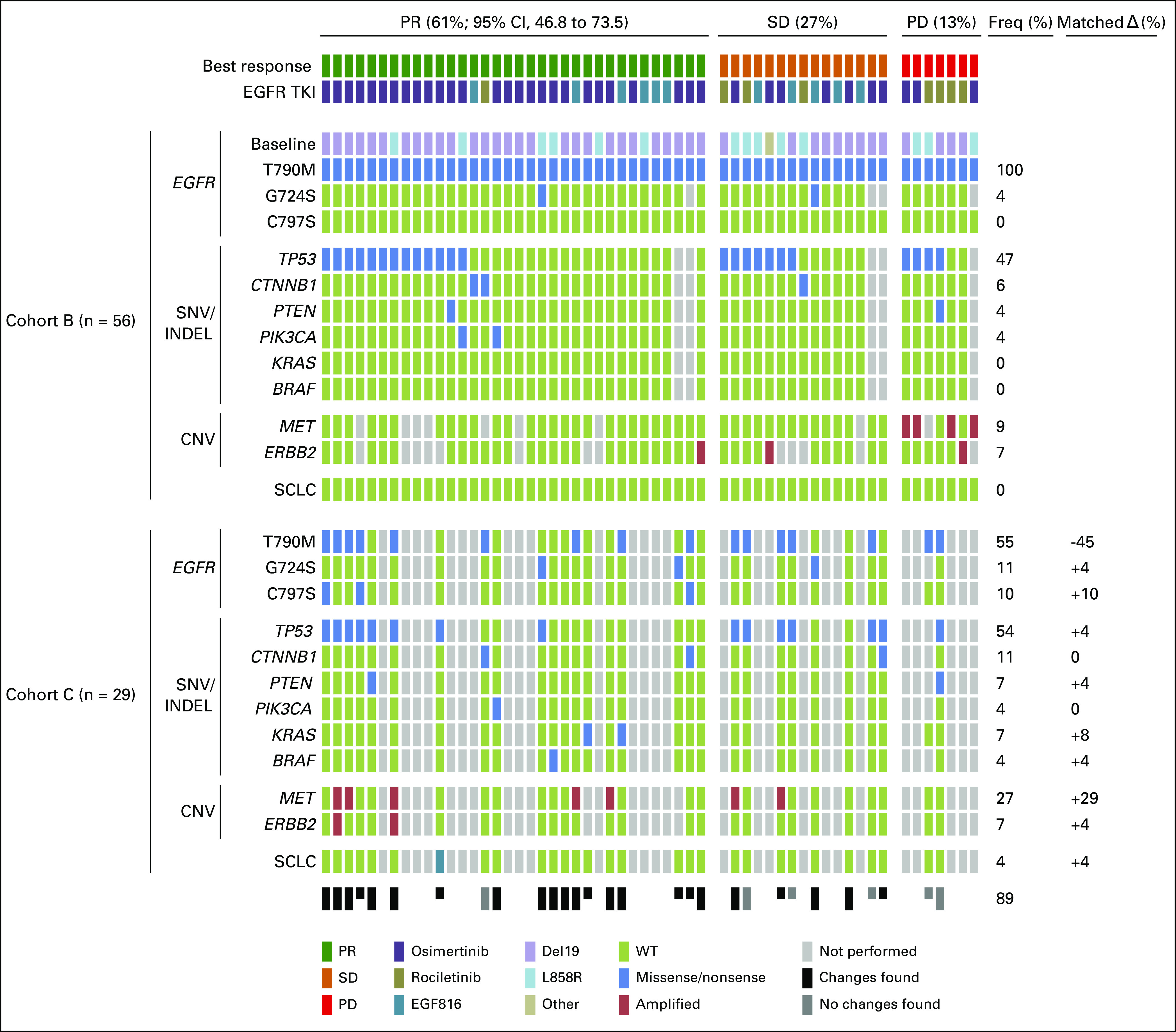
Map of genetic aberrations detected by sequencing (single nucleotide variant [SNV] and insertion/deletion [INDEL]) and copy number variation (CNV) analyses in biopsy specimens of epidermal growth factor receptor (*EGFR*) p.T790M-positive patients before treatment with a third-generation EGFR tyrosine kinase inhibitor (TKI; ie, osimertinib, rociletinib, nazartinib; upper block; cohort B; n = 56) and at progression to the specific treatment (lower block; cohort C; n = 29). The change in the frequency of specific aberrations during the course of treatment in matched samples is indicated in the lower block on the far right (Matched Δ). Half boxes indicate incomplete molecular work-up. Freq, frequencies; PD, progressive disease; PR, partial response; SCLC, small-cell lung cancer; SD, stable disease; WT, wild type.

Initial tumor stage, gender, smoking status, and the number of prior EGFR TKIs had no significant impact on treatment outcomes (Table 1). A map of molecular aberrations found in patients from cohort B is displayed in Figure 2 (Data Supplement). OS (47.0 months; 95% CI, 27.2 to 66.8 *v* 55.3 months; 95% CI, 48.9 to 61.7 months; *P* = .307), PFS (7.3 months; 95% CI, 1.3 to 13.3 *v* 8.1 months; 95% CI, 6.5 to 9.7 months; *P* = .354), and RR (54.2%; 95% CI, 32.8% to 74.5% *v*. 70.4%; 95% CI, 49.8% to 86.3%; *P* = .261) were not significantly different in patients with *TP53* mutations compared with patients with *TP53* WT (Table 1). Only one of three (33.3%) *ERBB2*-amplified patients responded to treatment (*P* = .552). PFS and OS were 4.2 months (95% CI, 0.4 to 8.0 months) and 26.6 months (95% CI, 9.6 to 43.6 months) for *ERBB2*-amplified patients compared with 8.0 months (95% CI, 6.7 to 9.3 months; *P* = .933) and 56.6 months (95% CI, 41.9 to 71.2 months; *P* = .825) in patients with *ERBB2* WT (Table 1). Similarly, in patients with mutations in *PTEN* and *PIK3CA,* OS, PFS, and RR were nonsignificantly reduced (Table 1).

**FIG 3.**
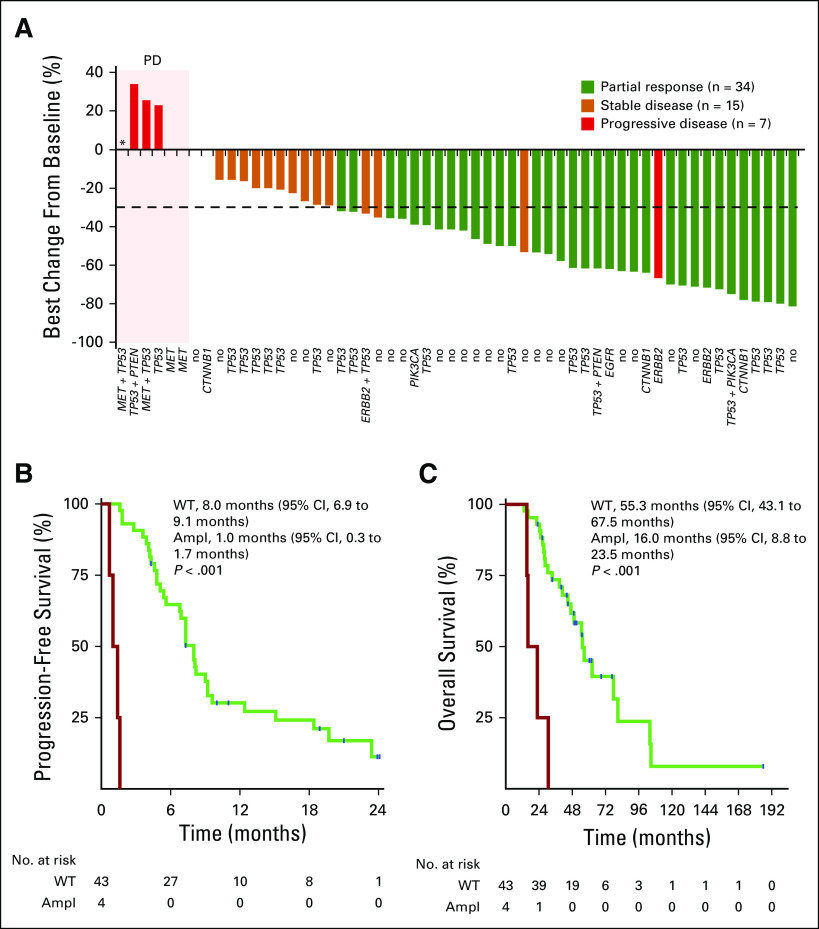
(A) Waterfall plot showing the best change in percent of the target lesions according to Response Evaluation Criteria in Solid Tumors (RECIST) 1.1 per patient during treatment with a third-generation epidermal growth factor receptor (EGFR) tyrosine kinase inhibitor (TKI; n = 56; cohort B). (*) Patient with progressive disease (PD) as best response but no target lesion measurement possible. Kaplan-Meier graphs displaying (B) progression-free survival and (C) overall survival for patients with *EGFR* p.T790M-positive non–small-cell lung cancer (NSCLC) with and without mesenchymal-epithelial transition factor (*MET*) amplification (ampl), who received treatment with third-generation EGFR TKIs. Both median overall survival and progression-free survival are dramatically reduced in the presence of *MET* amplifications. ERBB2, human epidermal growth factor receptor 2 gene; WT, wild type.

The RR in patients with *MET* amplifications (n = 4; 9%) was 0% (PD rate, 100%) compared with 62.8% in patients with no *MET* amplification (*P* = .027; Table 1; Fig 3; Data Supplement). Similarly, PFS (1.0 month; 95% CI, 0.3 to 1.7 *v* 8.0 months; 95% CI, 6.9 to 9.1 months; *P* < .001) and OS (16.0 months; 95% CI, 8.8 to 23.5 *v* 55.3 months; 95% CI, 43.1 to 67.5 months; *P* < .001) were significantly shorter in *MET*-amplified patients (Table 1; Fig 3).

### Mechanisms of AR to Third-Generation EGFR TKI Therapy (cohort C)

**FIG 4.**
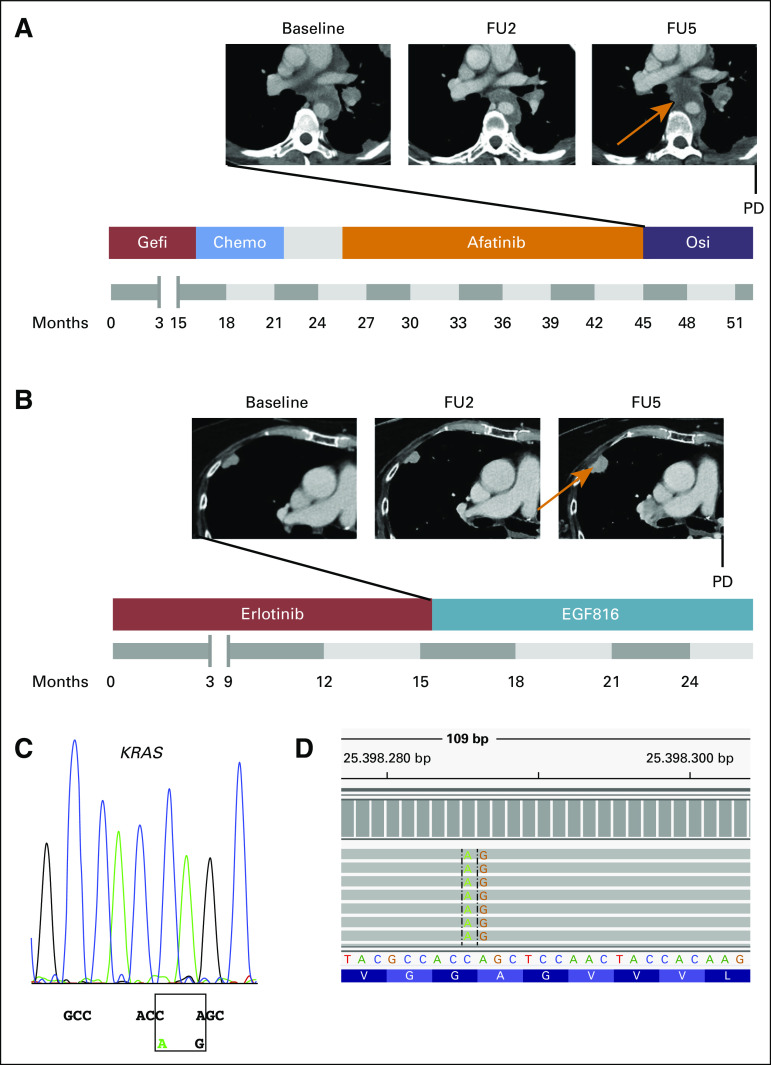
(A) Timeline showing the course of treatment of a female patient diagnosed with stage IV at 51 years of age. After treatment with gefitinib (gefi), platinum-doublet chemotherapy (chemo), and afatinib, the patient received osimertinib (osi; progression-free survival, 7.3 months). A progressive paraesophageal lesion was biopsied and revealed a *KRAS* p.G12S mutation and loss of p.T790M. The patient received local radiotherapy and died approximately 1.5 months later. (B) Timeline showing the course of treatment of a 76-year-old female patient initially diagnosed at stage II. Treatment with erlotinib was initiated once an epidermal growth factor receptor (*EGFR*) del19 was detected at recurrence of the disease. At progression, a p.T790M mutation was detected, and treatment with nazartinib was started, resulting in a good partial response. At progression, another biopsy at the spot indicated by the yellow arrow was collected, revealing a *KRAS* p.G12C mutation. (C) Analysis of the *KRAS* p.G12C mutation by Sanger sequencing. Electropherogram of the reverse sequencing reaction showing the nucleotide change c.33_34delinsCT. (D) Detection of the *KRAS* p.G12C mutation by massively parallel sequencing. The nucleotide change c.33_34delinsCT is visualized by the integrative genomics viewer. FU, follow-up; PD, progressive disease.

In total, 44 patients (79%) in cohort B had disease progression, and tumor samples were available from 29 patients (52%; cohort C; Figs 1 and 2). The results of the molecular analyses were matched with pretreatment samples and one earlier sample, if possible, to distinguish between passenger and acquired aberrations. The calculation of the frequency of changes in a gene compared with the pretreatment sample was performed in matched samples only (Fig 2; Data Supplement). The overall percentage of samples in which we detected acquired changes in the molecular pattern was 89% (n = 23). Loss of *EGFR* p.T790M was by far the most common molecular change (n = 13 of 29; 45%). Isolated loss of p.T790M without any other genetic change was detected in four samples (n = 4 of 26; 15%). However, we found small-cell lung cancer transformation in one sample (4%), which showed loss of p.T790M. Acquisition of high-level *MET* amplification was detected in seven samples (n = 7 of 25; 29%), and the mean *MET* copy number increased significantly between pretreatment and post-treatment biopsies (GCN mean, 2.8 *v* 6.3; two-tailed, pairwise *t* test *P* = .02; Data Supplement). The third most common genetic changes in cohort C were acquisition of *EGFR* p.C797S (n = 3 of 29; 10%), of which two were in *cis* and one in *trans* position, and loss of p.T790M with acquisition of p.G724S (n = 3 of 28; 11%). Amplification of *ERBB2* was observed in two samples (7%) and occurred together with *MET* amplification. Both patients were *MET* and *ERBB2* WT in pretreatment samples and had a long PFS of 15.1 and 19.7 months, respectively. Common *KRAS* mutations were detected in two samples (7%)—*KRAS* p.G12S and p.G12C. The *KRAS* p.G12C mutation involved the change of two consecutive nucleotides c.33_34delinsCT on the same allele, with an allelic fraction of 2.7%. Both patients are illustrated in Figure 4. Acquired mutations in *BRAF* (p.V600E), *TP53* (p.E180*), and *PTEN* (p.S229*) were detected in one sample each (4%). Mutations in *PIK3CA* and *CTNNB1* were already present in pretreatment samples in patients where matched samples were available and were considered as passenger mutations.

### Genetic Clustering of AR Mechanisms to Third-Generation EGFR TKIs and Impact on Third-Generation EGFR TKI Efficacy (cohort C)

**FIG 5.**
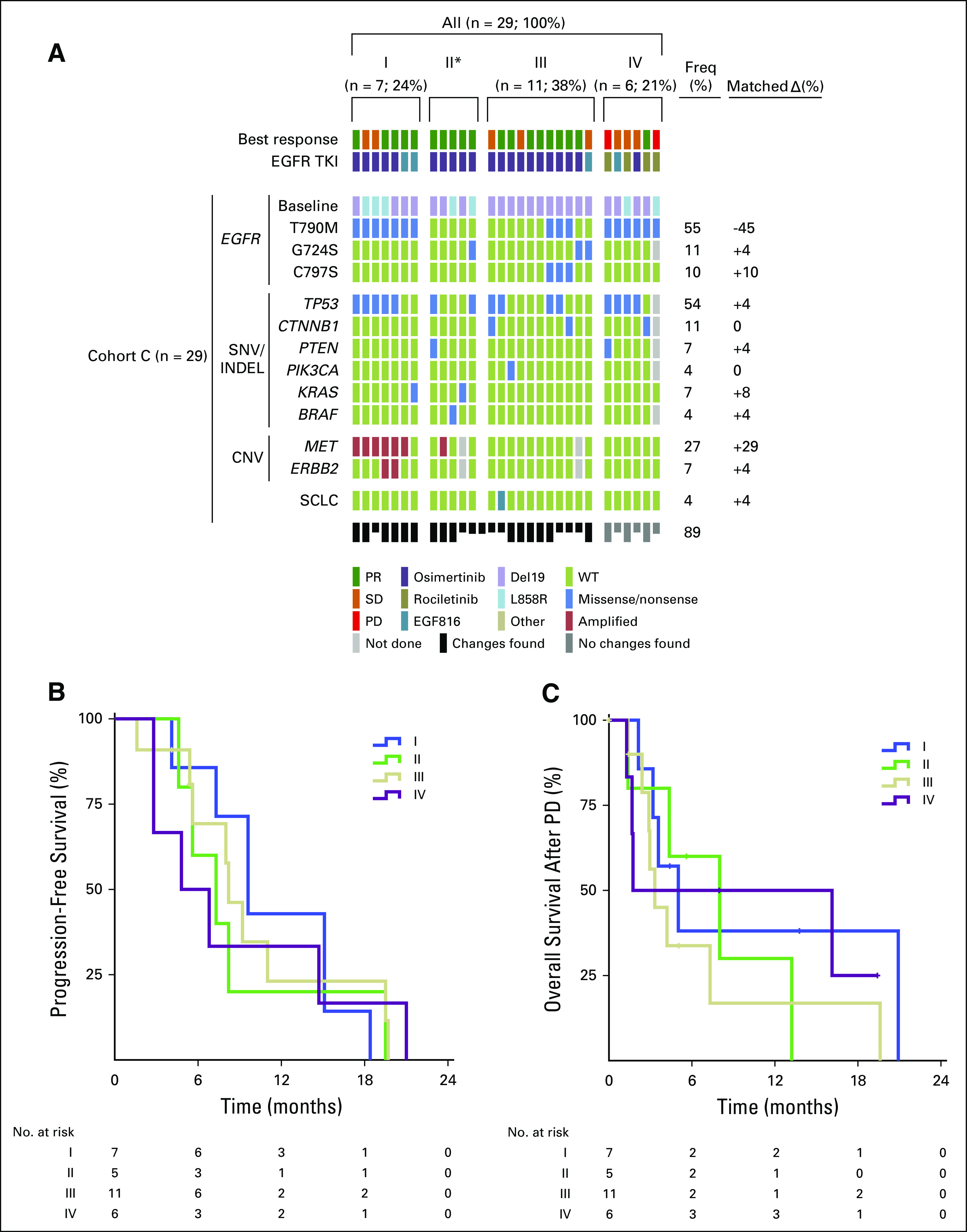
(A) Map of genetic aberrations detected by sequencing (single nucleotide variant [SNV] and insertion/deletions [INDELs]) and copy number variation (CNV) analyses in biopsy specimens collected after treatment with a third-generation epidermal growth factor receptor (EGFR) tyrosine kinase inhibitor (TKI; cohort C; n = 29). Patients were clustered in four groups: (I) changes outside of *EGFR* only, (II) changes in *EGFR* and outside of *EGFR*, (III) changes in *EGFR* only, and (IV) no changes found. The change in the frequency of specific aberrations during the course of treatment in matched samples is indicated in the lower block on the far right (Matched Δ). Half boxes indicate incomplete molecular work-up. (B) Progression-free survival of patients by cluster. Median progression-free survival (95% CI): I, 9.6 months (6.7 to 12.6 months); II, 7.3 (3.7 to 11.0 months); III, 8.2 (6.5 to 9.9 months); and IV, 4.8 (0.0 to 9.6 months). Levels of statistical significance for comparison of clusters were *P* > .1. (C) Overall survival by cluster from progressive disease (PD) on third-generation EGFR inhibitor treatment until death. Median overall survival (95% CI): I, 5 months (2.1 to 8.0 months); II, 8 (2.3 to 13.7 months); III, 3.3 (2.3 to 4.4 months); and IV, 1.7 (0.0 to 15.6 months). Levels of statistical significance for comparison of clusters were *P* > .1. *ERBB2*, human epidermal growth factor receptor 2 gene; freq, frequencies; MET, mesenchymal-epithelial transition factor; PR, partial response; SCLC, small-cell lung cancer; SD, stable disease; WT, wild type. (*) n = 5; 17%.

Occurrence of multiple mechanisms of AR followed a distinctive pattern (Fig 5A). Changes in *EGFR*, such as loss of p.T790M and acquisition of p.C797S, were mutually exclusive. Except for one patient, CNV in *MET* and/or *ERBB2* did not occur together with p.C797S or loss of p.T790M. In the samples with new *BRAF* and *TP53* mutations, as well as in one of the patients with *KRAS*-mutant disease, p.T790M was lost. *ERBB2* amplifications were all found in samples that also harbored amplifications of *MET*.

We therefore clustered the patients in four groups: (I) changes outside of *EGFR* only, (II) changes in *EGFR* and outside of *EGFR*, (III) changes in *EGFR* only, and (IV) no changes found (Fig 5A). Seven patients (24%) belonged to cluster I, and 11 belonged to cluster III (38%). Five patients (17%) had changes in and off the target at the same time (cluster II). No changes were found in six patients (21%; cluster IV). In patients treated with osimertinib, a larger fraction belonged to cluster III than cluster I or II (n = 10; 47.6% for III *v* n = 5; 23.8% for I and II). In patients treated with rociletinib, this trend was inversed (changes in *EGFR*, n = 0; 0% *v* no changes found, n = 4; 100%). Of the four patients treated with nazartinib, two (50%) displayed changes outside of EGFR. In one patient (25%), changes in *EGFR* were found. No changes were found in another patient (25%). The statistical significance for a cross table stratified by cluster and type of EGFR TKI was *P* = .002 (Fisher’s exact test). Differences in PFS by cluster were not statistically significant (Fig 5B). Similarly, OS after PD was also not significantly different between the clusters (Fig 5C). Overall response rate (ORR) was 71.4% (n = 5) for patients in cluster I, 100% (n = 5) in cluster II, 72.2% (n = 8) in cluster III, and 16.7% (n = 1) in cluster IV (Fisher’s exact test for comparison of all clusters, *P* = .022).

Nine patients (31%) received a treatment trying to match the targets identified in the molecular analysis. Median duration of treatment was 1.8 months (95% CI, 0.3 to 3.3 months) for targeted approaches versus 2.6 months (95% CI, 0.0 to 5.2 months) for chemotherapy (n = 4; *P* = .891; Data Supplement).

## DISCUSSION

Tumor heterogeneity turns out to be one of the key mechanisms underlying resistance to EGFR-targeted therapies.^17-19,21-28^ In this study, we analyzed pretreatment and post-treatment biopsy samples and clinical features of patients with NSCLC treated with third-generation EGFR inhibitors to assess determinants of IR and AR.

Our first analysis revealed a high genomic heterogeneity in patients with p.T790M-positive resistance to early-generation EGFR inhibitors. Some of these aberrations, for example, amplifications of *MET,* are known to cause AR to any EGFR TKI.^17-19^ The role of others, such as *TP53*, *PTEN*, *PIK3CA*, and *CTNNB1*, however, is still not well characterized.

 We therefore sought to determine the effect of these aberrations on third-generation EGFR TKI treatment outcomes. Overall efficacy and OS were similar in patients treated with osimertinib and nazartinib and in concordance with the data reported so far. However, patients treated with rociletinib had a worse outcome than reported previously, which may be caused by the low patient number. Several groups have reported on an association of *TP53* mutations and shorter OS in patients with *EGFR*-mutant NSCLC. However, most of these reports were not statistically significant, and similarly, OS, RR, and PFS were only numerically reduced in patients with *TP53* mutations in our study.^32-38^ Patient numbers with aberrations in *PTEN*, *PIK3CA*, and *ERBB2* were low, and the differences in treatment efficacy were not statistically significant. However, preclinical models and reports on small patient series suggest a negative impact of these aberrations on EGFR TKI therapy.^7,17,19,39,40^ In contrast, survival and treatment efficacy were dramatically impaired in patients with *MET*-amplified tumors, putting *MET* in the front line of potential mechanisms of IR.

To define mechanisms of AR to third-generation EGFR TKIs, we analyzed post-treatment biopsies of 29 patients (cohort C) and found that loss of p.T790M was by far the most frequent genetic change. However, only a small fraction of patients had an isolated loss of p.T790M. It is likely that other genetic changes that we did not detect with our analysis may contribute to AR in these patients with a loss of p.T790M and no other genetic change.^23^ The acquisition of p.C797S was detected in three patients, and several studies have confirmed the resistance-mediating effect of this substitution to osimertinib treatment.^23,41^ In addition, we found the secondary *EGFR* mutation p.G724S in three samples. In contrast to p.C797S, p.G724S was also in part detected in the samples collected at progression to early-generation EGFR TKIs.^29^^,^^42^ However, after failure of third-generation EGFR TKI treatment, p.G724S was always co-occurring with loss of p.T790M, suggesting the treatment-induced selection of this mutation. Acquisition of *MET* amplification was the second most frequent event associated with AR to third-generation EGFR inhibition, and similar frequencies have been described in the literature.^19,23^ The high prevalence of *MET* amplification in IR and AR points out the crucial role of *MET* in EGFR inhibitor resistance. Interestingly, amplifications of *MET* and *ERBB2* occurred together in two patients. It is unclear whether this reflects the existence of two independent tumor clones or whether both aberrations are acquired in the same clone and how they influence therapy outcome. We also found acquired mutations in *KRAS* in two patients and a *BRAF* p.V600E mutation in one patient. Activation of the MEK/extracellular regulated kinase pathway through *KRAS* mutations as an escape mechanism and efficacy of the combined EGFR and MEK inhibition was reported previously.^17,26,27^ Thus, taken together, treatment of *EGFR*-mutant NSCLC with TKIs targeting EGFR as well as MET and MEK may delay the development of AR and prevent IR in selected patients.

By clustering the genetic findings at AR into four groups—mechanism of resistance off target (I), on target (III), or in both (II), and no changes detected (IV)—we found a distinct molecular pattern depending on the EGFR TKI applied. Changes in *EGFR* were almost exclusively found in patients treated with osimertinib. In contrast, no patient treated with rociletinib displayed changes in *EGFR*, and other studies have confirmed the absence of secondary *EGFR* mutations in patients with progression while taking rociletinib.^19,43^ It is conceivable that this effect may be caused by a lower selection pressure of rociletinib on cells with on-target aberrations. We also found a statistically significant association between cluster and ORR, because patients in cluster IV had a markedly reduced ORR to third-generation EGFR treatment. However, differences in PFS or OS after PD were not significant.

In summary, our study first shows that molecular heterogeneity of p.T790M-mutant lung cancer with AR to early-generation EGFR TKIs influences efficacy of third-generation inhibitors. Our observations also show the need to integrate information on co-occurring alterations in the design of clinical trials, aiming at a more precise identification of patients who benefit from combined targeted treatment. Because osimertinib has been approved for first-line treatment of *EGFR*-mutant NSCLC in many countries, our analysis may be of relevance to a decreasing subgroup. But mechanisms of resistance to first-line osimertinib have not been well characterized, and it is conceivable that recurrent mechanisms of resistance to EGFR inhibition such as *MET* amplification, MET activation, and *EGFR* p.C797S may also play a major role in this setting.

## Data Availability

The following represents disclosure information provided by authors of this manuscript. All relationships are considered compensated. Relationships are self-held unless noted. I = Immediate Family Member, Inst = My Institution. Relationships may not relate to the subject matter of this manuscript. For more information about ASCO's conflict of interest policy, please refer to www.asco.org/rwc or ascopubs.org/po/author-center. **Honoraria:** Novartis, Pfizer, AstraZeneca, Boehringer Ingelheim, Roche Pharma AG **Consulting or Advisory Role:** Boehringer Ingelheim, Pfizer, Roche Pharma AG **Research Funding:** Pfizer (Inst), Novartis (Inst), Bristol-Myers Squibb (Inst) **Travel, Accommodations, Expenses:** Novartis **Honoraria:** AstraZeneca, Illumina **Other Relationship:** Pfizer **Consulting or Advisory Role:** Pfizer, Roche Molecular Diagnostics, MSD, AstraZeneca, AbbVie, Bristol-Myers Squibb **Speakers' Bureau:** Quadia **Research Funding:** AstraZeneca, Roche Molecular Diagnostics, Personal Genome Diagnostics **Travel, Accommodations, Expenses:** Takeda, Pfizer, Roche **Travel, Accommodations, Expenses:** Ventana Medical Systems **Honoraria:** Bristol-Myers Squibb **Travel, Accommodations, Expenses:** Novartis **Honoraria:** Genentech, Boehringer Ingelheim, Pfizer, AbbVie, MSD, Bristol-Myers Squibb, Ipsen, Novartis **Consulting or Advisory Role:** AstraZeneca, Genentech, Pfizer, Boehringer Ingelheim, MSD, Bristol-Myers Squibb, Celgene, Takeda, AbbVie, Novartis, Bayer **Research Funding:** AstraZeneca (Inst), Boehringer Ingelheim (Inst), Bristol-Myers Squibb (Inst), MSD (Inst), Celgene (Inst), Eli Lilly (Inst), Novartis (Inst), Pfizer (Inst), Roche (Inst), Takeda (Inst) **Honoraria:** AstraZeneca **Honoraria:** Bristol-Myers Squibb, Roche, MSD **Research Funding:** Bristol-Myers Squibb (Inst), MSD (Inst) **Travel, Accommodations, Expenses:** Mediolanum **Other Relationship:** AstraZeneca, Pfizer **Honoraria:** MSD Sharp & Dohme **Consulting or Advisory Role:** Novartis **Honoraria:** MSD Oncology, Boehringer Ingelheim, LEO Pharma, PharmaMar, Roche, Pfizer, Chugai Pharma, Takeda **Consulting or Advisory Role:** MSD Oncology, Bristol-Myers Squibb, Sanofi, Roche, AstraZeneca **Travel, Accommodations, Expenses:** Sanofi, Eli Lilly, Amgen, Novartis, PharmaMar, Boehringer Ingelheim, MSD Oncology, Bristol-Myers Squibb **Consulting or Advisory Role:** AstraZeneca (Inst), Bristol-Myers Squibb (Inst), MSD Oncology (Inst), Roche (Inst), Boehringer Ingelheim (Inst), Pfizer (Inst) **Research Funding:** AstraZeneca (Inst), Bristol-Myers Squibb (Inst), Merck Serono (Inst), MSD Oncology (Inst), Roche (Inst) **Honoraria:** Roche, Bristol-Myers Squibb, Merck Sharp & Dohme, Pfizer, Boehringer Ingelheim **Consulting or Advisory Role:** Bristol-Myers Squibb, F. Hoffmann-La Roche, Merck Sharp & Dohme, Pfizer, Boehringer Ingelheim **Speakers' Bureau:** F. Hoffmann-La Roche, Bristol-Myers Squibb, Boehringer Ingelheim **Research Funding:** Merck Serono **Travel, Accommodations, Expenses:** Bristol-Myers Squibb, F. Hoffmann-La Roche, MSD Oncology, Boehringer Ingelheim **Honoraria:** Pfizer, Celgene, Novartis, Roche, Boehringer Ingelheim, Janssen, Bristol-Myers Squibb **Consulting or Advisory Role:** Novartis, Boehringer Ingelheim, Bristol-Myers Squibb, Roche, Janssen, Pfizer **Research Funding:** Pfizer, (Inst), Bristol-Myers Squibb (Inst), Novartis (Inst), MSD (Inst), Janssen (Inst) **Travel, Accommodations, Expenses:** Novartis, Pfizer, Celgene, Boehringer Ingelheim **Stock and Other Ownership Interests:** Roche, Foundation Medicine **Patents, Royalties, Other Intellectual Property:** A patent of NRG1 fusions has been filed **Consulting or Advisory Role:** MSD **Travel, Accommodations, Expenses:** Shire **Consulting or Advisory Role:** Amgen, Celgene **Honoraria:** MSD, Bristol-Myers Squibb, Roche, Dako/Agilent Technologies **Consulting or Advisory Role:** MSD, Bristol-Myers Squibb, Roche, Dako/Agilent Technologies **Honoraria:** Healthcare Consulting Cologne, Boehringer Ingelheim, Takeda **Consulting or Advisory Role:** Boehringer Ingelheim, Takeda **Travel, Accommodations, Expenses:** Boehringer Ingelheim **Honoraria:** Novartis, Celgene, Roche, Bristol-Myers Squibb, Ipsen, Pfizer, AstraZeneca **Consulting or Advisory Role:** MSD, Pfizer **Travel, Accommodations, Expenses:** Astellas Pharma, Celgene, Ipsen **Honoraria:** AstraZeneca, Novartis, Pfizer/EMD Serono, MSD, Takeda, Bristol-Myers Squibb, Eli Lilly, Genentech, Boehringer Ingelheim, AbbVie **Consulting or Advisory Role:** Genentech, MSD, AstraZeneca, AbbVie, Takeda, Eli Lilly, Boehringer Ingelheim, Novartis, Bristol-Myers Squibb, Pfizer, Celgene **Travel, Accommodations, Expenses:** Pfizer, Takeda **Research Funding:** Novartis, Novartis **Employment:** NEO New Oncology, Hämatopathologie Hamburg **Honoraria:** Roche Pharma, AstraZeneca, Bristol-Myers Squibb, Boehringer Ingelheim **Consulting or Advisory Role:** Roche Pharma, Bristol-Myers Squibb, Novartis **Consulting or Advisory Role:** Pfizer, Roche, Boehringer Ingelheim, AstraZeneca, Bristol-Myers Squibb, Celgene, Guardant Health, Novartis, Takeda, AbbVie, Blueprint Medicines, Eli Lilly, Merck KGaA, Merck Sharp & Dohme **Speakers' Bureau:** AstraZeneca, Bristol-Myers Squibb, Novartis, Boehringer Ingelheim, Merck Sharp & Dohme, Roche, Pfizer, AbbVie, Eli Lilly, Merck KGaA, Takeda **Research Funding:** Fundación Merck Salud (Inst), EMD Serono (Inst) **Honoraria:** AstraZeneca, Bristol-Myers Squibb, Novartis, Pfizer, Roche Pharma **Consulting or Advisory Role:** Bristol-Myers Squibb, Novartis, Pfizer **Consulting or Advisory Role:** Eli Lilly, AstraZeneca (Inst), Boehringer Ingelheim (Inst), Genentech (Inst), Bristol-Myers Squibb (Inst), Merck KGaA (Inst), MSD Oncology (Inst), Takeda (Inst), Bayer (Inst) **Research Funding:** Boehringer Ingelheim (Inst), Bayer (Inst), Genentech (Inst), AstraZeneca (Inst), Bristol-Myers Squibb (Inst) **Stock and Other Ownership Interests:** Co-founder and CSO for Targos Mol. Pathol. (Kassel/Germany) and TAMP (Atlanta, GA) **Honoraria:** AstraZeneca, AbbVie, Bayer, Bristol-Myers Squibb, Boehringer Ingelheim, Merck Serono, MSD, Novartis, Qiagen, Pfizer, Roche **Research Funding:** Roche (Inst) **Honoraria:** AbbVie, AstraZeneca, Bristol-Myers Squibb, Boehringer Ingelheim, MSD, Novartis, Roche **Consulting or Advisory Role:** AbbVie, AstraZeneca, Bristol-Myers Squibb, Boehringer Ingelheim, Chugai Pharma, Ignyta, Eli Lilly, MSD Oncology, Novartis, Pfizer, Roche **Research Funding:** Bristol-Myers Squibb, Novartis, Pfizer No other potential conflicts of interest were reported.
